# Remedy for Cough

**Published:** 1844-01

**Authors:** 


*Remedy for Cough.*—The abrupt violation of the decencies of social existence is one of the most annoying consequences of coughing and sneezing. If you happen to have a catarrh, and are not endowed with sufficiently strong powers of mind to be able to resist a paroxysm of coughing, place the end of your fore-finger upon the end of your nose, if you have a nose. In the absence of this central organ, rub your eyes as if you had been magnetized, and look at your watch. If the fit of coughing does not pass off, you will at least pass for an accomplished gentleman.—*Med. Chi. Rev.*, October, 1843.

